# Supplementary material for: Antibody fine specificity correlates with protection from malaria for the RTS,S vaccine in young African children: A post hoc analysis of a phase IIb randomised controlled trial
Source: PLoS Med. 2026 May 14;23(5):e1004877. doi: 10.1371/journal.pmed.1004877 (PMC13186370; doi:10.1371/journal.pmed.1004877)
Supplement: S1 Appendix — Table A. Overview of published studies examining the association between IgG to NANP-repeats and protection from malaria among infants and young children residing in malaria-endemic countries immunised with the RTS,S vaccine. Table B. Cox proportional-hazard (CPH) model hazard ratios (HR) for NANP15, NANP2 and J1 IgG in RTS,S vaccinated children adjusted by age (aHR). Fig A. Study design and statistical analysis approach. A) Summary of the preclinical and clinical samples tested for IgG to six CSP-specific peptides, indicating what data are included in each figure. B) The causal framework for analysis to identify correlates of protection (outcome) in RTS,S vaccinated children (exposure) is demonstrated through a Directed Acyclic Graph (DAG). Age and sex were considered as potential confounders, a priori, but only age was associated with vaccine responses and was included in the final adjusted model. No other confounders were identified. Fig B. IgG to NANP15, NANP3 and NANP2 peptides in a subset of children vaccinated with RTS,S. A subset of serum samples (n = 40, study month 3 time point) from children who received RTS,S in a phase IIb clinical trial were A) tested for IgG to NANP15, NANP3 and NANP2 at a 1/500 dilution by ELISA. The OD median (centre line) and interquartile range (whiskers) of each group is shown. B) IgG data for each peptide is also shown on a scatterplot with the Spearman’s correlation coefficient (rho) and 95% confidence interval (CI), corresponding p-values shown. Fig C. IgG to NANP15, NANP2 and J1 peptides by P. falciparum exposure (merozoite IgG) in RTS,S vaccinated children. Vaccinated children (n = 715) were tested for IgG binding to merozoites by ELISA as a biomarker of P. falciparum exposure. The IgG responses to merozoites were categorised as high (n = 357) and low (n = 358) exposure based on the group median OD = 0.220. The median of the high exposure group was OD = 0.442 (range, 0.220,1.206) and of the low exposure group was OD = 0.100 [file pmed.1004877.s001.pdf]

S1 Appendix

Supplementary methods

Animal immunisations.

We evaluated IgG specificity to peptides representing various CSP epitopes in mice vaccinated with the same truncated form of CSP as the RTS,S vaccine, expressed as a virus-like particle (VLP) using the duck hepatitis B surface antigen (ARTES Biotechnology GmbH) as previously described [1]. For comparison, we also evaluated IgG responses in mice vaccinated with recombinant full-length CSP [2]. To increase the sample size, mice were pooled from two vaccine studies. In study 1, Swiss mice received three fortnightly doses of RTS,S (n=5, 10µg per dose) or CSP (n=4, 10µg per dose) with alum adjuvant, and we evaluated sera collected two weeks after the final vaccination. In study 2, C57BL/6 mice received two monthly doses of RTS,S (n=5, 3µg per dose, ARTES Biotechnology GmbH) or CSP (n=4, 5µg per dose, produced in-house) with Quil-A adjuvant, and we evaluated sera collected 24 or 38 weeks after the final vaccination (respectively) [3]. We also assessed sera collected prior to vaccination as a negative control to determine a seropositivity threshold of optical density (OD)=0.1 (based on mean IgG + 2 standard deviations (SD) response to each peptide). To investigate antibody specificity following vaccination with the major NANP-repeat motif (and not the entire RTS,S construct), we also vaccinated mice with VLPs expressing only the NANP-repeats as previously described [4]. Briefly, BALB/c mice received four doses of VLPs expressing no antigen as a negative control (n=7, 1µg per dose), $NANP_4$ (n=7, 1µg per dose) or $NANP_9$ (n=7, 1µg per dose) with CpG adjuvant. We evaluated sera collected two weeks after the final vaccination. Animal immunisations and collections were performed at Walter and Eliza Hall Institute (WEHI, AU) animal facility (ethics approval number: 2020.019).

Antibody detection by enzyme-linked immunosorbent assay.

We evaluated IgG binding to each CSP peptide by enzyme-linked immunosorbent assay (ELISA) as previously described [5]. Flat bottom 96-well Nunc MaxiSorp plates (#442404, ThermoFisher) were coated in 1µg/mL streptavidin (#S4762, Sigma-Aldrich; 50µL per well) overnight at 4°C. The plates were blocked with 0.1% casein in phosphate-buffered saline (PBS, w/v, 200µL per well) for 2 hours at 37°C and then incubated with 0.5µg/mL biotinylated peptide (50µL per well) for 1 hour at 4°C. Note that for the peptides that were not biotinylated ($NANP_{15}$ or Junction-repeat) 0.5µg/mL of peptide was

directly coated onto 96-well plates (50µL per well) overnight at 4°C (without streptavidin), and blocked with 0.1% casein-PBS the following day. Serum samples were tested in duplicate and diluted in 0.1% casein-PBS (50µL per well) for 2 hours at room temperature. Mouse samples were tested at a 1/100 or 1/4000 dilution and IgG was detected using goat anti-mouse IgG conjugated to horseradish peroxidase (HRP) at 1/1000 in 0.1% casein-PBS (#AP308P, Sigma-Aldrich, Darmstadt, Germany). The mAbs were tested between 0.001µg/mL and 2µg/mL and detected by a two-step method using mouse anti-human IgG1 HRP at 1/1000 (#A10630, Invitrogen, Massachusetts, USA), followed by goat anti-mouse IgG HRP at 1/1000 (#AP308P, Invitrogen). RTS,S pooled samples were tested between a 1/250 and 1/64000 dilution. Individual RTS,S samples were tested at a 1/1000 dilution for IgG to $NANP_{15}$ and a 1/500 dilution for IgG to $NANP_2$, J1 and Junction-repeat peptides. RTS,S-induced IgG were detected using goat anti-human IgG HRP at 1/1000 in 0.1% casein-PBS (#62-8420, Invitrogen, Massachusetts, USA). Finally, 2,2'-azino-bis (3-ethylbenzothiazoline-6-sulfonic acid) (ABTS) (#00-2024, Sigma-Aldrich) or tetramethylbenzidine (TMB) (#00-2023, Sigma-Aldrich) substrate was added to plates (50µL per well) and shielded from light. If TMB was used, the reaction was stopped using 1M H_2SO_4 (50µL per well). Plates were read at 405nm for ABTS or 450nm for TMB substrates (MultiSkan Go, ThermoFisher Scientific), and the results were obtained as optical density (OD) values. In between each step, the plates were washed three times using PBS-Tween 0.05% (v/v). Raw data were corrected for background reactivity using no-sample control wells and adjusted for plate-to-plate variation using positive controls.

The ELISA procedure was slightly modified in some instances. Given the large number of individual RTS,S samples, we used high-throughput ELISA to detect IgG in RTS,S individual serum samples. High protein binding 384-well plates (#6007500, SpectraPlate, Perkin Elmer) were used, reagents were dispensed using a multidrop dispense (40µL per well for all steps, except for blocking which was 80µL per well), and samples were added using the Janus Automated Workstation (Perkin Elmer) as previously described [5]. To detect IgG binding to *P. falciparum* merozoites, 96-well plates were coated in merozoites (50,000 per well, in PBS) and incubated for 2 hours at 37°C. *Plasmodium falciparum* D10 (FC27 clone) parasites were cultured and merozoites were isolated as previously described [6-8]. Following coating, wells were blocked in 10% skim milk (in PBS, w/v) at 37°C for 2 hours. Sera and goat anti-human IgG HRP were diluted in 5% skim milk (in PBS, w/v). Finally, TMB was added and left to incubate for 30 minutes in the dark for colour development. We also assessed avidity using established methods in the presence of ammonium thiocyanate, as previously described [6].

Immunoassay to detect functional antibody responses.

We evaluated C1q complement fixation by adapting the standard ELISA protocol, as previously described [5]. Antigen coating and blocking were performed as above and following the serum step, purified human C1q was added (10μg/mL, 30μL per well, #C1740, Merck) for 30min at room temperature. To detect C1q fixation to the Junction-repeat peptide, individual RTS,S serum samples were tested at a 1/100 dilution factor. Rabbit anti-C1q (1/2000, 50μL per well, produced in-house) was used, followed by goat anti-rabbit IgG HRP (1/2000, 50μL per well, #1706515, Biorad). Lastly, TMB substrate and H_2SO_4 were used (50μL per well each). We also measured FcγRIII binding to the Junction-repeat peptide using previously described protocols [5]. RTS,S sera were tested at a 1/500 dilution factor, and we used 1% bovine serum albumin (BSA) in PBS (w/v) instead of casein in PBS to block and dilute reagents. Following serum incubation, a biotin-conjugated, human recombinant FcγRIII was added (0.1μg/mL, 50μL per well, produced in-house) and left to incubate for 1 hour at 37°C. Then, following washing, HRP-conjugated streptavidin (#21130, ThermoFisher Scientific) was added to the plate (1/10,000 dilution factor, 50μL per well) and left to incubate for 1 hour at 37°C. TMB substrate and H_2SO_4 were used (50μL per well each).

Supplementary tables

S1 Table A. Overview of published studies examining the association between IgG to NANP-repeats and protection from malaria among infants and young children residing in malaria-endemic countries immunised with the RTS,S vaccine. The table only includes IgG to NANP-repeats as this is the current reference assay (other immune parameters are not included here).

**Vaccine trial**	**Population**	**Correlate of protection**
RTS,S/AS02, Phase I/IIb	Infants 10 weeks, Mozambique	Yes, against infection [9]
RTS,S/AS02, Phase IIb	Infants 8 weeks, Tanzania	Yes, against infection [10]
RTS,S/AS02, Phase IIb	Children 1-4 years, Mozambique	Yes, against infection [11]
		No, against malaria [5, 11, 12]
RTS,S/AS01, Phase II	Infants 6-10 weeks, multiple African sites	Yes, against malaria [13, 14]
	Children 5-17 months, Kenya and Tanzania	No, against malaria [14, 15]
RTS,S/AS01, Phase III	Infants 6-10 weeks, multiple African sites	Yes, against malaria [16]
	Children 5-17 months, multiple African sites	No, against malaria [16]
	Infants and children, multiple African sites	Yes, against malaria [17]
		No, against malaria [18]

S1 Table B. Cox proportional-hazard (CPH) model hazard ratios (HR) for $NANP_{15}$, $NANP_2$ and J1 IgG in RTS,S vaccinated children adjusted by age (aHR).

Antigen	Response	HR [95% CI]	p-value	[c]aHR [95% CI]	p-value
[a]$NANP_{15}$	IgG	0.984 [0.841, 1.152]	0.845	0.903 [0.765, 1.065]	0.225
[a]$NANP_2$	IgG	0.523 [0.729, 0.997]	0.046	0.838 [0.716, 0.981]	0.028
[a]J1	IgG	0.769 [0.656, 0.901]	0.001	0.718 [0.611, 0.844]	<0.001
[b]$NANP_2$+J1	IgG	0.523 [0.373, 0.735]	<0.001	0.470 [0.332, 0.666]	<0.001

[a] Optical density (OD) was normalised to the standard deviation (SD) and used in CPH models as a continuous variable (n=646).

[b] OD was used to determine high (H) and low (L) responders (based on median OD); only those who were high (HH) or low (LL) for both antigens were included in CPH models (n=474).

[c] Adjusted for age.

Supplementary data

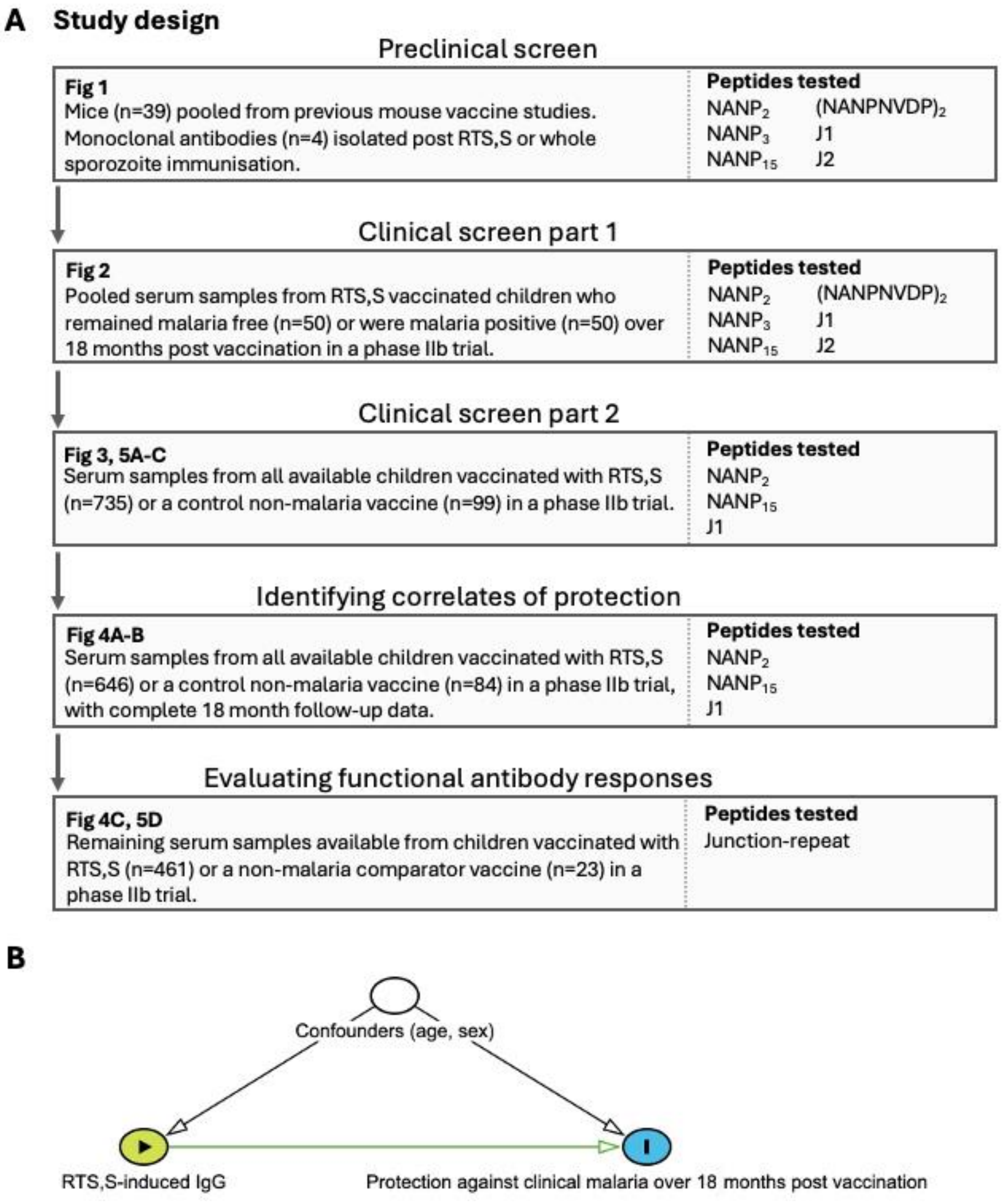

S1 Fig A. Study design and statistical analysis approach. A) Summary of the preclinical and clinical samples tested for IgG to six CSP-specific peptides, indicating what data are included in each figure. **B)** The causal framework for analysis to identify correlates of protection (outcome) in

RTS,S vaccinated children (exposure) is demonstrated through a Directed Acyclic Graph (DAG). Age and sex were considered as potential confounders, *a priori*, but only age was associated with vaccine responses and was included in the final adjusted model. No other confounders were identified.

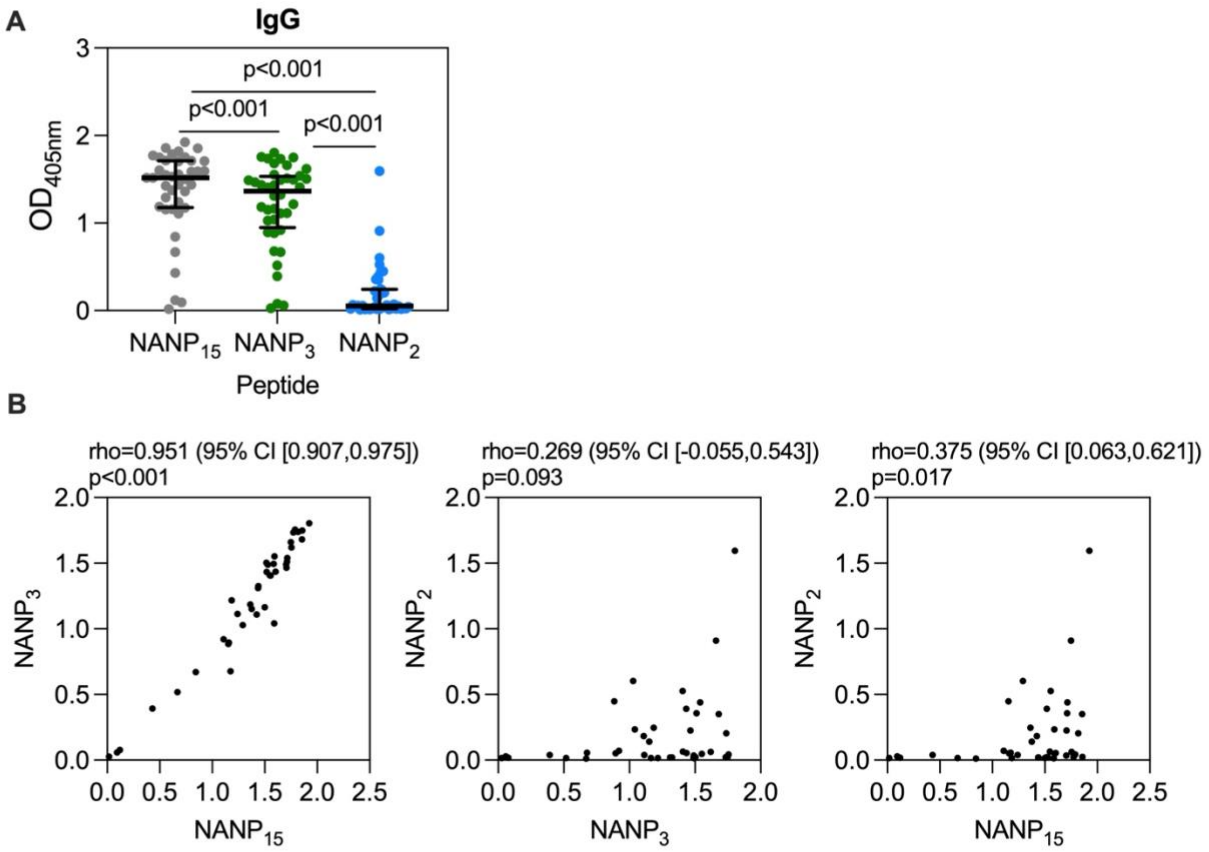

S1 Fig B. IgG to $NANP_{15}$, $NANP_3$ and $NANP_2$ peptides in a subset of children vaccinated with RTS,S. A subset of serum samples (n=40, study month 3 timepoint) from children who received RTS,S in a phase IIb clinical trial were **A)** tested for IgG to $NANP_{15}$, $NANP_3$ and $NANP_2$ at a 1/500 dilution by ELISA. The OD median (centre line) and interquartile range (whiskers) of each group is shown. **B)** IgG data for each peptide is also shown on a scatterplot with the Spearman's correlation coefficient (rho) and 95% confidence interval (CI), corresponding p-values shown.

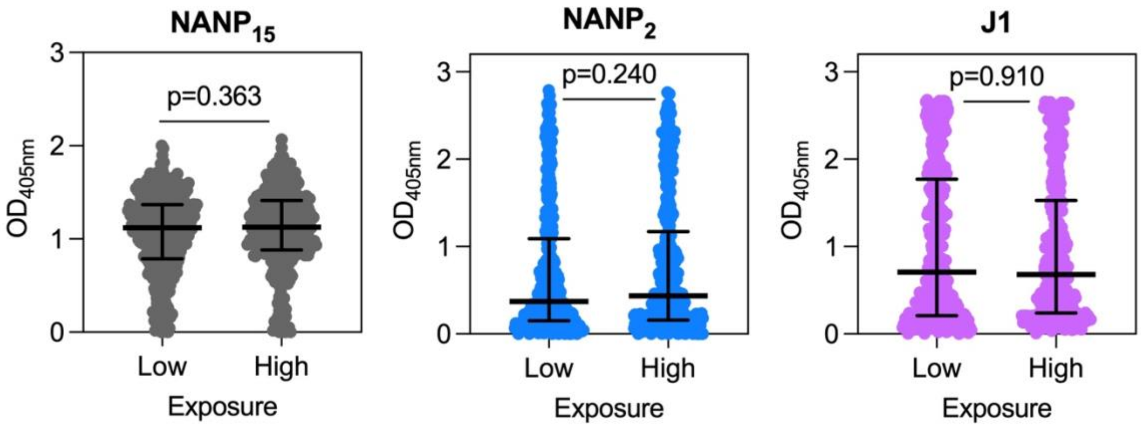

S1 Fig C. IgG to NANP$_{15}$, NANP$_2$ and J1 peptides by *P. falciparum* exposure (merozoite IgG) in RTS,S vaccinated children. Vaccinated children (n=715) were tested for IgG binding to merozoites by ELISA as a biomarker of *P. falciparum* exposure. The IgG responses to merozoites were categorised as high (n=357) and low (n=358) exposure based on the group median OD=0.220. The median of the high exposure group was OD=0.442 (range, 0.220,1.206) and of the low exposure group was OD=0.100 (range, 0.000,0.200). Vaccine IgG responses to NANP$_{15}$, NANP$_2$ and J1 are shown for high versus low exposure groups. The OD median (centre line) and interquartile range (whiskers) of each group is shown. The Mann-Whitney U test was performed to compare between groups and corresponding p-values are shown.

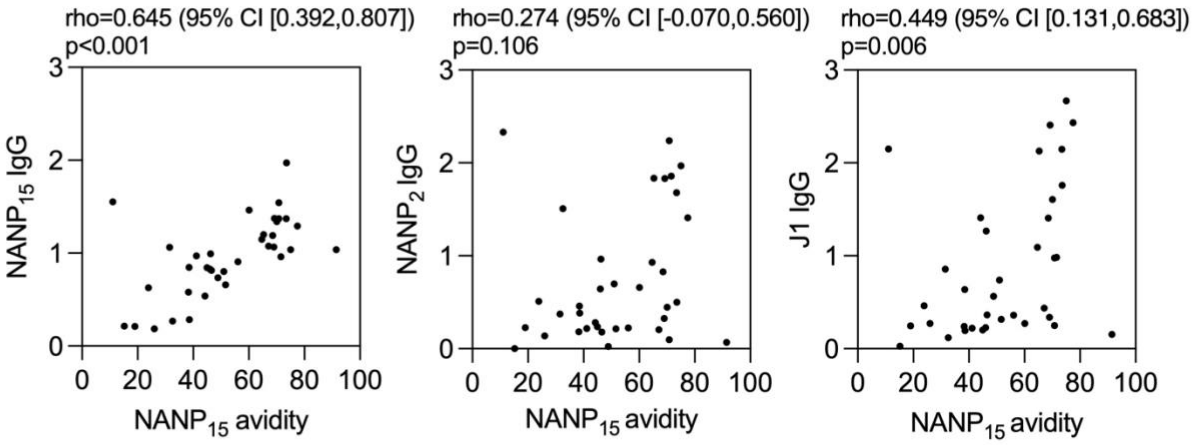

S1 Fig D. $NANP_{15}$, $NANP_2$, and J1 IgG correlation with $NANP_{15}$ avidity. Sera from a subset of RTS,S vaccinated children (n=36, study month 3 timepoint) were tested for antibody avidity to $NANP_{15}$ IgG and the data was correlated with IgG to $NANP_{15}$, $NANP_2$, and J1 peptides and shown on a scatterplot. The Spearman's correlation coefficient (rho) is shown with 95% confidence interval (CI), corresponding p-values are shown.

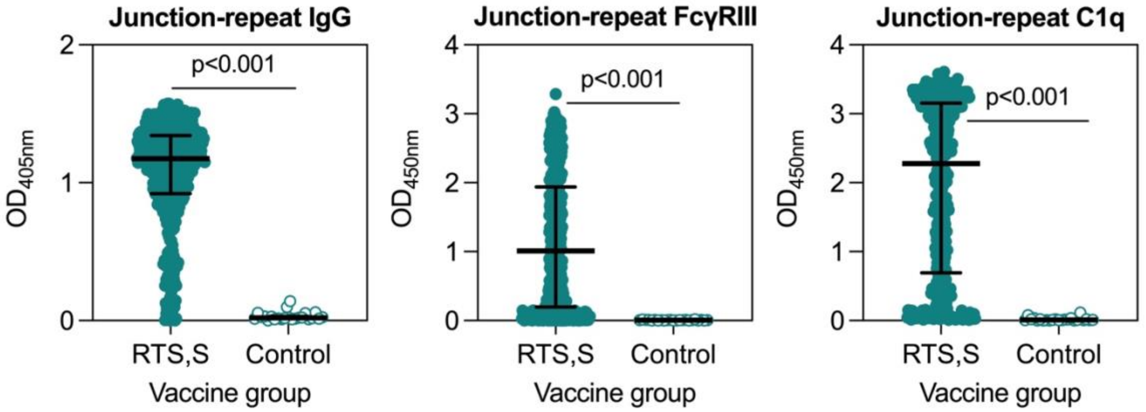

S1 Fig E. IgG and functional antibody responses to the Junction-repeat peptide comprising NANP, NPDP and NVDP motifs in a subset of children immunised with RTS,S or a control non-malaria vaccine. Serum samples were collected from children who received three doses of RTS,S (n=461) or a control non-malaria vaccine (n=23) at study month 3 timepoint. Sera were tested in duplicate for Junction-repeat-IgG at a 1/500 dilution factor, Junction-repeat-FcγRIII binding at a 1/500 dilution factor and Junction-repeat-C1q fixation at a 1/100 dilution factor by ELISA. The OD median (centre line) and interquartile range (whiskers) of each group is shown. The Mann-Whitney U test was performed to compare between vaccine groups and corresponding p-values are shown.

References

1. Kurtovic L, Wetzel D, Reiling L, Drew DR, Palmer C, Kouskousis B, et al. Novel Virus-Like Particle Vaccine Encoding the Circumsporozoite Protein of *Plasmodium falciparum* Is Immunogenic and Induces Functional Antibody Responses in Mice. Frontiers in Immunology. 2021;12.
2. Kastenmüller K, Espinosa DA, Trager L, Stoyanov C, Salazar AM, Pokalwar S, et al. Full-length *Plasmodium falciparum* circumsporozoite protein administered with long-chain poly(I·C) or the Toll-like receptor 4 agonist glucopyranosyl lipid adjuvant-stable emulsion elicits potent antibody and CD4+ T cell immunity and protection in mice. Infect Immun. 2013;81(3):789-800.
3. Kurtovic L, Drew DR, Dent AE, Kazura JW, Beeson JG. Antibody Targets and Properties for Complement-Fixation Against the Circumsporozoite Protein in Malaria Immunity. Frontiers in Immunology. 2021;12.
4. Kingston NJ, Kurtovic L, Walsh R, Joe C, Lovrecz G, Locarnini S, et al. Hepatitis B virus-like particles expressing *Plasmodium falciparum* epitopes induce complement-fixing antibodies against the circumsporozoite protein. Vaccine. 2019;37(12):1674-84.
5. Kurtovic L, Feng G, Hysa A, Haghiri A, O'Flaherty K, Wines BD, et al. Antibody mechanisms of protection against malaria in RTS, S-vaccinated children: a post-hoc serological analysis of phase 2 trial. The Lancet Microbe. 2024;5(10).
6. Boyle MJ, Chan JA, Handayuni I, Reiling L, Feng G, Hilton A, et al. IgM in human immunity to *Plasmodium falciparum* malaria. Science Advances. 2019;5(9):eaax4489.
7. Beeson JG, Brown GV, Molyneux ME, Mhango C, Dzinjalamala F, Rogerson SJ. Plasmodium falciparum isolates from infected pregnant women and children are associated with distinct adhesive and antigenic properties. The Journal of infectious diseases. 1999;180(2):464-72.
8. Osier FH, Feng G, Boyle MJ, Langer C, Zhou J, Richards JS, et al. Opsonic phagocytosis of Plasmodium falciparum merozoites: mechanism in human immunity and a correlate of protection against malaria. BMC medicine. 2014;12(1):108.
9. Aponte JJ, Aide P, Renom M, Mandomando I, Bassat Q, Sacarlal J, et al. Safety of the RTS, S/AS02D candidate malaria vaccine in infants living in a highly endemic area of Mozambique: a double blind randomised controlled phase I/IIb trial. The Lancet. 2007;370(9598):1543-51.
10. Abdulla S, Oberholzer R, Juma O, Kubhoja S, Machera F, Membi C, et al. Safety and immunogenicity of RTS, S/AS02D malaria vaccine in infants. New England Journal of Medicine. 2008;359(24):2533-44.
11. Guinovart C, Aponte JJ, Sacarlal J, Aide P, Leach A, Bassat Q, et al. Insights into long-lasting protection induced by RTS, S/AS02A malaria vaccine: further results from a phase IIb trial in Mozambican children. PloS one. 2009;4(4):e5165.
12. Alonso PL, Sacarlal J, Aponte JJ, Leach A, Macete E, Milman J, et al. Efficacy of the RTS,S/AS02A vaccine against *Plasmodium falciparum* infection and disease in young African children: randomised controlled trial. Lancet. 2004;364(9443):1411-20.
13. Asante KP, Abdulla S, Agnandji S, Lyimo J, Vekemans J, Soulanoudjingar S, et al. Safety and efficacy of the RTS, S/AS01E candidate malaria vaccine given with expanded-programme-on-immunisation vaccines: 19 month follow-up of a randomised, open-label, phase 2 trial. The Lancet Infectious Diseases. 2011;11(10):741-9.
14. Olotu A, Lusingu J, Leach A, Lievens M, Vekemans J, Msham S, et al. Efficacy of RTS, S/AS01E malaria vaccine and exploratory analysis on anti-circumsporozoite antibody titres and protection in children aged 5–17 months in Kenya and Tanzania: a randomised controlled trial. The Lancet Infectious Diseases. 2011;11(2):102-9.

15. Bejon P, Lusingu J, Olotu A, Leach A, Lievens M, Vekemans J, et al. Efficacy of RTS, S/AS01E vaccine against malaria in children 5 to 17 months of age. New England Journal of Medicine. 2008;359(24):2521-32.
16. Rts S. Efficacy and safety of RTS, S/AS01 malaria vaccine with or without a booster dose in infants and children in Africa: final results of a phase 3, individually randomised, controlled trial. Lancet. 2015;386(9988):31-45.
17. Dobaño C, Sanz H, Sorgho H, Dosoo D, Mpina M, Ubillos I, et al. Concentration and avidity of antibodies to different circumsporozoite epitopes correlate with RTS, S/AS01E malaria vaccine efficacy. Nature communications. 2019;10(1):2174.
18. Ubillos I, Ayestaran A, Nhabomba AJ, Dosoo D, Vidal M, Jiménez A, et al. Baseline exposure, antibody subclass, and hepatitis B response differentially affect malaria protective immunity following RTS, S/AS01E vaccination in African children. BMC medicine. 2018;16(1):197.
