# Supplementary material for: Antibody fine specificity correlates with protection from malaria for the RTS,S vaccine in young African children: A post hoc analysis of a phase IIb randomised controlled trial
Source: PLoS Med. 2026 May 14;23(5):e1004877. doi: 10.1371/journal.pmed.1004877 (PMC13186370; doi:10.1371/journal.pmed.1004877)
Supplement: S3 Checklist — Information on the STROBE initiative is available at https://www.strobe-statement.org/, licensed under the Creative Commons Attribution 4.0 International (CC BY 4.0). The STROBE checklist originates from von Elm E et al. 2008. J Clin Epidemiol, 61(4) and can be accessed on https://doi.org/10.1016/j.jclinepi.2007.11.008. (PDF) [file pmed.1004877.s005.pdf]

STROBE Statement—checklist of items that should be included in reports of observational studies

	Item No	Recommendation	Section/paragraph number
**Title and abstract**	1	(*a*) Indicate the study's design with a commonly used term in the title or the abstract	Title and Abstract "methods and findings" section.
		(*b*) Provide in the abstract an informative and balanced summary of what was done and what was found	Abstract "methods and findings" and "conclusions" sections.
**Introduction**			
Background/rationale	2	Explain the scientific background and rationale for the investigation being reported	Introduction, paragraphs 1-3.
Objectives	3	State specific objectives, including any prespecified hypotheses	Introduction, paragraph 4.
**Methods**			
Study design	4	Present key elements of study design early in the paper	Methods section.
Setting	5	Describe the setting, locations, and relevant dates, including periods of recruitment, exposure, follow-up, and data collection	Methods "Study participants" section.
Participants	6	(*a*) *Cohort study*—Give the eligibility criteria, and the sources and methods of selection of participants. Describe methods of follow-up<br>*Case-control study*—Give the eligibility criteria, and the sources and methods of case ascertainment and control selection. Give the rationale for the choice of cases and controls<br>*Cross-sectional study*—Give the eligibility criteria, and the sources and methods of selection of participants	Methods "Study participants" section.
		(*b*) *Cohort study*—For matched studies, give matching criteria and number of exposed and unexposed<br>*Case-control study*—For matched studies, give matching criteria and the number of controls per case	N/A.
Variables	7	Clearly define all outcomes, exposures, predictors, potential confounders, and effect modifiers. Give diagnostic criteria, if applicable	Methods section, 'Statistical analysis'.
Data sources/ measurement	8*	For each variable of interest, give sources of data and details of methods of assessment (measurement). Describe comparability of assessment methods if there is more than one group	Methods "Immunoassays to detect antibody binding and functional responses" section.
Bias	9	Describe any efforts to address potential sources of bias	Methods section.
Study size	10	Explain how the study size was arrived at	Methods "Study participants" section.

Quantitative variables	11	Explain how quantitative variables were handled in the analyses. If applicable, describe which groupings were chosen and why	Methods “Study participants” and “Statistical analysis” sections.
Statistical methods	12	(*a*) Describe all statistical methods, including those used to control for confounding	Methods “Statistical analysis” section.
		(*b*) Describe any methods used to examine subgroups and interactions	Methods “Statistical analysis” section.
		(*c*) Explain how missing data were addressed	Methods.
		(*d*) *Cohort study*—If applicable, explain how loss to follow-up was addressed<br>*Case-control study*—If applicable, explain how matching of cases and controls was addressed<br>*Cross-sectional study*—If applicable, describe analytical methods taking account of sampling strategy	N/A.
		(*e*) Describe any sensitivity analyses	N/A.

Continued on next page

Results

Participants	13*	(a) Report numbers of individuals at each stage of study—eg numbers potentially eligible, examined for eligibility, confirmed eligible, included in the study, completing follow-up, and analysed	Methods "Study participants" section.
		(b) Give reasons for non-participation at each stage	Methods "Study participants" section.
		(c) Consider use of a flow diagram	Methods "Study participants" section.
Descriptive data	14*	(a) Give characteristics of study participants (eg demographic, clinical, social) and information on exposures and potential confounders	Methods "Study participants" section.
		(b) Indicate number of participants with missing data for each variable of interest	Methods "Study participants" section.
		(c) *Cohort study*—Summarise follow-up time (eg, average and total amount)	Methods "Study participants" section.
Outcome data	15*	*Cohort study*—Report numbers of outcome events or summary measures over time	Methods "Study participants" section.
		Case-control study—Report numbers in each exposure category, or summary measures of exposure	N/A.
		Cross-sectional study—Report numbers of outcome events or summary measures	N/A.
Main results	16	(*a*) Give unadjusted estimates and, if applicable, confounder-adjusted estimates and their precision (eg, 95% confidence interval). Make clear which confounders were adjusted for and why they were included	Methods "Statistical analysis" section; Results "IgG specificity and cross-reactivity are associated with protection against clinical malaria in young children vaccinated with RTS,S" section; "Supplementary Tables "Table S2" and Results.
		(*b*) Report category boundaries when continuous variables were categorized	Results.
		(*c*) If relevant, consider translating estimates of relative risk into absolute risk for a meaningful time period.	N/A.
Other analyses	17	Report other analyses done—eg analyses of subgroups and interactions, and sensitivity analyses	All analyses are described in the Methods and Results section.

**Discussion**			
Key results	18	Summarise key results with reference to study objectives	Discussion, paragraph 1.
Limitations	19	Discuss limitations of the study, taking into account sources of potential bias or imprecision. Discuss both direction and magnitude of any potential bias	Discussion, paragraph 7.
Interpretation	20	Give a cautious overall interpretation of results considering objectives, limitations, multiplicity of analyses, results from similar studies, and other relevant evidence	Discussion paragraphs 1-6.
Generalisability	21	Discuss the generalisability (external validity) of the study results	Discussion, paragraph 7.
**Other information**			
Funding	22	Give the source of funding and the role of the funders for the present study and, if applicable, for the original study on which the present article is based	"Financial disclosure statement" section.

*Give information separately for cases and controls in case-control studies and, if applicable, for exposed and unexposed groups in cohort and cross-sectional studies.

Note: An Explanation and Elaboration article discusses each checklist item and gives methodological background and published examples of transparent reporting. The STROBE checklist is best used in conjunction with this article (freely available on the Web sites of PLoS Medicine at http://www.plosmedicine.org/, Annals of Internal Medicine at http://www.annals.org/, and Epidemiology at http://www.epidem.com/). Information on the STROBE Initiative is available at www.strobe-statement.org.
