# Supplementary material for: Antibody fine specificity correlates with protection from malaria for the RTS,S vaccine in young African children: A post hoc analysis of a phase IIb randomised controlled trial
Source: PLoS Med. 2026 May 14;23(5):e1004877. doi: 10.1371/journal.pmed.1004877 (PMC13186370; doi:10.1371/journal.pmed.1004877)
Supplement: Attachment — Submitted filename: Hysa et al. Response to reviewer comments_02.03.26.pdf [file pmed.1004877.s006.pdf]

Antibody fine specificity reveals new correlates of protection for the RTS,S malaria vaccine in young African children

RESPONSE TO REVIEWER COMMENTS

Please note that all page and line numbers refer to the revised clean manuscript file and supplementary file which are now two separate documents.

Reviewer #1

The development and evaluation of malaria vaccine based on an immune response to the CSP protein has been hindered by the lack of a reliable serological correlate of protection. Thus, the findings of this paper that antibody titres to a short NANP repeat and to a peptide at the junctional region of the CSP antigen provide a stronger correlation of protection than the ELISA used in most previous studies is important and could aid future vaccine development based on the CSP antigen, including RNA vaccine. This is an important finding. The investigators describe progression through a logical series of approaches to demonstration of the association between vaccine induced responses to these antigen and protection against clinical episode of malaria in young African children. The paper is well written, and results including the figures presented clearly so I have only a few comments and suggestions.

Methods

Monoclonal antibodies: It is not clear why the specific monoclonal antibodies tested were selected for these studies. Could this be explained?

RESPONSE: In our study, we selected several mAbs that have been previously characterised and were known to recognise the major NANP-repeat motif so we could assess potential binding to diverse peptide sequences that included minor repeat and junction epitopes. We included mAbs derived from humans immunised with RTS,S (mAbs 311 and 317) or live-attenuated sporozoites (mAb MGG4) as this may influence antibody fine specificity. We also included mAb 2A10, which was originally isolated many decades ago and is one of the most

widely used anti-CSP mAbs in *in vitro* (and *in vivo*) studies. These provided a representative selection of mAbs relevant to our questions and to confirm the performance of our assays. Furthermore, the sequences of these mAbs had been previously published, enabling us to express them in-house for our study. We have elaborated on these points in the methods section (page 8, lines 161-165).

Sera from the phase 2 trial in Mozambique: These samples are now 20 years old, and I wonder if they have been thawed and frozen on previous occasions for other studies. Were original aliquots used?

RESPONSE: We were provided sample aliquots as part of an ancillary study to evaluate immunological responses induced by vaccination. These sample aliquots (in cryovials) have been appropriately stored at -80 °C at Burnet Institute and had minimal freeze/thawing to assess antibody responses. We are confident in the quality of the samples given the high reactivity observed in the vaccine group relative to that of the control group. Serum/plasma is known to be stable in long-term storage at -70/80 °C and freeze-thawing cycles have limited impact on antibodies. For example, for SARS CoV 2 IgG, up to 16 freeze–thaw cycles had no significant effect on ELISA OD values or qualitative positivity in serum (Shurrab et al 2021, PMID: 34356000). We have conducted similar tests for antimalarial antibodies and similarly found no effect.

Statistical analysis: Was a formal SAP developed before the results from the Mozambiquan study were analysed?

RESPONSE: A statistical analysis plan was in place for this study. The aim was to evaluate antibodies associated with risk of malaria during follow-up using the Cox-proportional hazards model with time to first event. This followed the same analysis approach used in the original trial assessing vaccine efficacy (time to first event using Cox proportional hazards model) as referenced in the methods (page 10, line 222-226). Reflecting on the reviewer's comments, we decided it would be helpful for readers to have an overview of our study approach and analysis – we have now provided a flow chart and overview as Supplementary Figure S1 (page 6, line 87). Part A summarises our approach and the data that is included in

each figure. Part B of the figure shows the causal framework for analysis as a directed acyclic graph (DAG).

Ethics: Ethics for the clinical trial was obtained from the appropriate ethics committees at the time of the study. Did the consent form include provision for retention of the samples for this long period and for their use in subsequent laboratory studies?

RESPONSE: Yes, all relevant ethics approvals were attained and provided to the Human Research and Ethics Committee at the Alfred Hospital, who reviewed and approved the use and storage of samples at Burnet Institute for the current study (page 7, lines 137-141).

Results

I am not qualified to comment on the details of the immunological assays that were used but these are primarily assays that have been well validated previously and conducted by the authors. Results are presented clearly and were easily followed including the figures.

Murine studies: The numbers of mice included in the different groups was generally very small for this kind of study. Was there a reason for this?

RESPONSE: We used the mouse studies to identify differences in antibody fine specificity following vaccination with full-length CSP, truncated CSP that represented the RTS,S vaccine, or different lengths of the NANP major repeat motif. We previously performed these vaccine studies and found our sample size was sufficient to detect differences in antibody magnitude between the different vaccine groups (Kurtovic, Wetzel et al., 2021; Kingston et al., 2019). In the present study, these sample sizes were sufficient to demonstrate reactivity to different peptides and detect major differences in antibody fine specificity, which we observed for some peptides (Figure 1). Our study had between 7-10 mice per vaccine group, which is similar to many other published mouse studies (e.g., Ryan et al., 2025; Krenger et al., 2024). Because the mice are inbred, housed under the same conditions, and have the same exposure and timing (vaccination), there is much less variance than seen in human populations. Small group sizes of 5-6 mice are very common in mouse studies.

Combination of J1 and NANP2: Was the combination associated with a significantly greater level of protection against clinical malaria than either peptide given alone.

RESPONSE: We would like to clarify that none of the participants were given any peptides; the samples were collected from children who received the RTS,S malaria vaccine as part of a phase 2b clinical trial. We evaluated the fine specificity of vaccine-induced antibody responses to individual peptides, including J1 and $NANP_2$. The adjusted HR for malaria risk was lower for children with antibodies to both peptides than for individual peptides (Figure 4b, Supplementary Table S2), but the confidence intervals for the respective HRs were overlapping so we must be cautious in interpreting the differences. Additional studies in other RTS,S vaccine cohorts using our assays will help resolve this question more confidently.

Discussion

Full length peptide: Do the investigators have an explanation of why this long peptide does not provide a more effective immune response?

RESPONSE: It remains unclear why antibodies to long NANP major repeat sequences poorly correlate with protection in RTS,S and R21 vaccine studies (reviewed in Beeson et al., Sci Transl Med 2022). The data presented in our paper provide some further insights. Our data shows that only a subset of children have antibodies with specificity for $NANP_2$ and/or junction peptides, and having those antibodies was strongly associated with protection. Therefore, quantifying antibodies to a long NANP-repeat sequence used in RTS,S vaccine trials does not discriminate children with different antibody specificities or children with protective and non-protective responses. In response to the reviewer's comments, we have included a short comment in the discussion (page 18-19, lines 434-439).

Heterogeneity in response: A striking feature of many serological studies conducted in African children vaccinated with RTS,S or R21, as in this study, is the wide range of response in the anti-CSP antibody response to the vaccine. Age was shown to be a factor in this study, with older children doing less well than younger ones, as observed in the R21 phase 3 trial, but gender and overall malaria exposure were not. Is this heterogeneity likely to involve

genetic factors? Do the authors have any other ideas as to what could account for this heterogeneity and are they undertaking any further studies to investigate this?

RESPONSE: Yes, we hypothesise that vaccine heterogeneity could be due to a range of potential genetic and environmental factors. In this cohort, it was not possible to study genetic factors due to ethical restrictions. However, we have future studies planned to address some of these questions in additional cohorts of RTS,S vaccinated children. We have added a comment on genetic factors to the discussion (page 20, lines 473-474).

Reviewer #2

This was a nicely written paper with interesting findings. It would be good to include the transmission rates/seasonality in Mozambique (and the specific area the study was conducted) at the time of the study.

RESPONSE: We have included these details in the methods section (page 8, lines 170-172).

Overall, the rationale for the use of 2,3 and 15 repeats of NANP needs a more robust justification, especially when many of the papers cited (and many in the field use) NANP with 6 repeats. Do the authors have data on IgG responses with NANP6? This would be an important addition.

RESPONSE: Native CSP contains approximately 37 NANP repeats, which can be difficult to express as recombinant protein or to synthesise as a peptide. Therefore, most studies of RTS,S vaccine responses have used polypeptides of multiple NANP repeats. We have extensively used the $NANP_{15}$ peptide to assess antibody responses to the central repeat region of CSP in naturally-acquired and vaccine-induced immunity (e.g., Kurtovic et al., BMC Med 2018, BMC Med 2019, J Infect Dis 2021, Front Immunol 2021). We previously showed that IgG to $NANP_{15}$ did not correlate with protection in RTS,S vaccinated children in this cohort (Kurtovic et al., Lancet Microbe 2024). This was consistent with an independent study that tested the same samples for IgG to R32LR (antigen that contains 30 NANP and 2 NVDP repeats and has been widely used in RTS,S trials) and also found no correlation with protection (Alonso et al. 2004 and Guinovart et al. 2009, summarised in Supplementary table S1). Some studies have used a $NANP_6$ peptide, though no clear rationale has been provided for using this construct.

Our main interest was to identify the shortest number of repeats that could be recognised by RTS,S-induced antibodies, and whether antibodies recognised junction epitopes that are not included in the RTS,S vaccine construct, and then examine whether responses to these epitopes were associated with protection. Previous work with mAbs suggested a peptide length of around 1.5 to 2.5 NANP repeats is the minimal epitope for antibody binding (Oyen

et al., 2017). Therefore, we synthesised the $NANP_2$ and $NANP_3$ peptides for our current study and quantified antibodies to them and found the former (but not the latter) could discriminate between malaria free and malaria positive pools (Figure 2).

To better illustrate this and further help readers understand our findings, we have included some additional analysis on antibodies to $NANP_2$, $NANP_3$, and $NANP_{15}$ among vaccinated children (Supplementary Figure S2), and described these results in the revised manuscript (page 13, lines 299-308). Our data show that only a subset of children have antibodies that can recognise the $NANP_2$ peptide, whereas nearly all children have antibodies to $NANP_3$ or $NANP_{15}$ peptides. IgG to $NANP_3$ and $NANP_{15}$ are very strongly correlated ($rho=0.951$; $p<0.001$), demonstrating that they largely measure the same response type. Therefore, we predict that antibodies to $NANP_6$ would be very similar to that of $NANP_3$ and $NANP_{15}$ and would not add any value to our study.

We have added additional text to explain our rationale for the use of these peptides in the methods (page 9, lines 195-197) and reported the findings in the results section (page 13, lines 299-308).

Its important to describe the reasons why mice were vaccinated with NANP4, NANP9 and NANP19 (RTS,S), but ELISAs developed for NANP2, NANP3 and NANP15. Why the difference?

RESPONSE: We hope that our earlier explanations, above, about the rationale behind using peptides of different lengths in assays of antibody specificity helps explain this. For the vaccination studies, we investigated whether vaccines based on different constructs of CSP and its regions generated antibodies to different epitopes, and second, whether vaccinating with different immunogens influences antibody fine specificity. We evaluated antibodies from mice vaccinated with full length CSP, an RTS,S-like vaccine, and vaccine that only included NANP repeats. We leveraged existing samples from a mouse vaccine study we previously conducted and published (Kingston et al., Vaccine 2019). The mouse vaccine study was originally designed to assess a novel vaccine construct whereby the NANP repeats were incorporated within the hepatitis B surface antigen rather than fused to the N-

terminus (which is the structure of RTS,S and R21), and contained a shorter NANP-repeat sequence than R21/RTS,S and did not include the CSP C-terminal domain that is present in RTS,S/R21. This was a good opportunity to assess how differences in NANP repeat numbers could potentially influence antibody find specificity, and we found that the longer $NANP_9$ vaccine was better at generating antibodies that cross-reacted with junction peptide sequences. Future studies could further investigate vaccine designs including different NANP repeat lengths, minor repeats and junction sequences, as we have considered in the discussion, but this is beyond the focus of the current manuscript.

The timepoints of clinical samples used is not specified and is needed.

RESPONSE: We evaluated samples collected 30 days after the third vaccine dose (three vaccine doses is the current primary vaccination regiment), which is study month 3 timepoint. These details have been added in the methods section (page 8, lines 182-183) and stated in the figure legends.

More information and justification is needed for the choice of clinical samples for various assays (not consistent between assays).

RESPONSE: We tested all available samples from children given the RTS,S vaccine (735 of 803; 92%) for IgG to $NANP_2$, J1 and $NANP_{15}$ peptides. We also tested a random selection of samples from children given the control vaccine (n=99) as it was previously established that antigen-specific antibodies were very low in this group (Alonso et al., Lancet 2004). We then evaluated functional antibody responses in all samples that had sufficient volume for these experiments. We have now included further details in the methods section (page 8-9, lines 183-193) and added Supplementary Figure S1A which gives an overview of the study and data that are included in each figure to help clarify this. We have checked and revised any wording in the manuscript to ensure this is clear.

The use of the word "validation" for assays in clinical immunology is quite specific and I don't think that the use of mAbs here satisfies what is generally considered to be an assay validation (https://urldefense.com/v3/__https://www.ema.europa.eu/en/ich-q2r2-validation-analytical-procedures-scientific-guideline__;!!G5ONXFxL7-

lpCg!bammr1Ps_PV1bFFBLjPCSvpY3B4ic0s-qmEMSVu134r-7LW7Ts5BER60IQU03U2WVsSnBuYfMuXVUEXVkQ8$). Suggest re-wording.

RESPONSE: For clarity, we have removed the term 'validated' because it is used in specific contexts on page 6 (line 131), page 11 (line 244), page 12 (line 285) and page 17 (line 404-405).

Please add justification for running functional assays using an amino acid sequence that is not used in ELISAs (the data for which is used as justification for running the functional assays).

RESPONSE: Antibody responses that function to fix complement and engage Fcγ-receptors require immune complex formation, or clustering of IgG molecules bound to antigens. Therefore, it is not currently possible to detect these functional activities using short peptides. For this reason, the peptides were only designed to assess antibody fine specificity of IgG binding and not functional activity. To overcome these limitations, we produced a peptide representing the sequence in CSP that comprises the junction peptide, minor repeat and major repeat sequences (see Table 1 and Figure 1A), which includes the epitopes of interest and was long enough to coat onto plates and accommodate antibody clustering for functional activity. We found that children with high IgG to both $NANP_2$ and J1 had higher functional responses, suggesting these epitopes were important. We have considered this in the discussion and noted that future studies to better understand the relative importance of antibodies targeting specific epitopes, or combinations, for functional activities would be valuable (page 19, lines 456-460 and page 21, lines 509-512).

Additionally, the functional assay references cited do not use the same sequence as used here. Please be more transparent about the differences and possible consequences of using different amino acid sequences for antigens in previous work by the group (and in references cited) and in the different quantification vs functionality work in this paper. This would be a good opportunity to highlight that the field in general is not very aligned and there is a pressing need for harmonisation (given the finding here of NANP2 (but not 3 or 15) being associated with vaccine-induced protection).

RESPONSE: The novelty of our study is that we evaluated antibody responses to antigens representing specific epitopes of CSP that have not been measured before in African children. We have previously published methods to detect antibody function against the $NANP_{15}$ peptide (e.g., Kurtovic et al., Lancet Microbe 2024), which were identical to the methods used in the current study (apart from the coating antigen used, as specified in the methods (page 9, lines 208-211) and results (page 15, lines 358-360)).

We agree that the field is not aligned in terms of antigens and assays used to assess vaccine immunogenicity. This is partly due to a lack of a defined correlate of protection. Our study findings are valuable because they identify better correlates of protection and encourage the quantification of IgG responses to $NANP_2$ and the junction sequence in future vaccine immunogenicity studies in the field. Considering the reviewer's comments, we have made a comment on this in the discussion, noting that future harmonisation of assays will be valuable for the field (page 20, lines 481-483). We have also reviewed our text and made edits where required to ensure our text is as clear as possible.

For the functional assays, it would be interesting to see these responses to either a longer NANP construct or an otherwise lengthened/stabilised (perhaps with BSA?) J1 epitope to overcome the issue of peptide length. The solution here of using a compound peptide is that it's not known which epitope the functional response is directed to.

RESPONSE: As noted above, there are technical limitations to measuring functional antibodies to short peptides. However, we agree that novel approaches to do this should be pursued in future studies. We currently do not have an established approach for this, but one approach that could be explored would be to present epitopes on a flexible scaffold. Developing such assays would be a substantial investment and is beyond the scope of the current study.

The papers cited on Fc-mediated activity of post-immunisation IgG (refs 43,44,45), seem like odd choices. Neither of the Behet papers measure FcR binding, the 2018 one looks at complement deposition, and the 2014 looks at inhibition of hepatocyte traversal but both using whole sporozoites incubation with post-immunisation IgG.

RESPONSES: We acknowledge the reviewer's comment and have revised the references accordingly. We removed citations for the Behet papers on page 15 (line 359) and page 17 (line 400).

Were there any genetic differences between individuals who did not get any episodes of malaria? Was this investigated as part of this study?

RESPONSE: This is an interesting point, but unfortunately host genetic testing was not performed in this clinical trial due to ethical restrictions. Samples for genetic testing were not collected, consent was not provided for genetic testing, and we do not have ethics approval for genetic testing. This should be investigated in future studies with appropriate sampling and consent. We have added a comment on genetic factors to the discussion (page 20, lines 473-474).

Specific line items:

Line 41: Should use updated 2024 stats.

RESPONSE: We have now included the most recent available statistics (for 2023) published in the WHO 2024 World Malaria Report (page 4, line 72).

Line 69: 19 repeats in RTS,S but 18 in R21

RESPONSE: According to the original R21 publication (Collins et al. 2017), R21 contains 19 NANP repeats in its construct and RTS,S is reported to also include 19 NANP repeats (Gordon et al. 1995) (references included in page 5, line 101).

Line 138 - were all available samples representative of the whole? Why were only this number available? What was the rate of malaria in the unvaccinated group? Please add compound peptide junction sequence used for functional assays into table 1

RESPONSE: We tested all available samples from children who received all three doses of RTS,S in this trial (735/803; 92%) (page 8, lines 176-178). We also tested a random selection of samples from the control vaccine group (n=99) as it has been previously shown that antigen-specific antibodies were very low in this group. In the control group, there were 159

recorded events per 302.9 person-years at risk within the 6-month follow-up period for the primary case definition as described in the original clinical trial (Alonso et al., Lancet 2004). We have added this to the method section (page 8, lines 175-176). The Junction-repeat peptide used for functional assays is already shown in Table 1.

-Line 169: They used a parametric stats test for preclinical results and non-parametric for clinical—is this conventional?

RESPONSE: Throughout the manuscript we used statistical tests that were appropriate for the data being analysed. We performed parametric statistical testing for the preclinical studies as the mice were genetically identical and raised in identical conditions and therefore assumed to have a normal data distribution. In contrast, we performed non-parametric statistical testing for the clinical studies as vaccine responses in African children were generally not normally distributed as has been previously shown (e.g., Kurtovic et al., Lancet Microbe 2024).

Line 173 "low and high responders" based in what? Which antigen?

RESPONSE: The text was referring to children being low and high responses to $NANP_{15}$, $NANP_2$ and J1 peptides which has been specified accordingly (page 10, lines 219-220).

Line 198: The mice were vaccinated using a homemade 'RTS,S' which is different to Mosquirix. How reliable is this?

RESPONSE: RTS,S is not commercially available for us to purchase and use in mouse vaccination studies. Therefore, we used the exact same antigenic construct expressed as virus-like particles using the duck Hepatitis B surface antigen, as specified in the methods (page 7, lines 143-146). We have previously shown this vaccine induces antibodies that are similar to human monoclonal antibodies derived from RTS,S vaccinated humans, which supports our construct being similar to that of RTS,S (Kurtovic, Wetzel et al., Front Immunol 2021).

Line 204 - add detail on seropositivity analysis

RESPONSE: The seropositivity threshold was calculated as OD=0.1 based on the overall mean + 2SD response of pre-vaccine samples to each peptide and has been described in the results (page 11, lines 258-259) and in Supplementary methods (page 1, lines 13-14).

Line 207 - were the proportions all overlapping?

RESPONSE: We described the seropositivity of mice to individual peptides, which is a binary measure (yes, no) determined as described above. In Figure 1, we have also presented individual antibody responses (continuous measure) along with the group mean and standard deviation.

Line 213 why did they decide to vaccinate with nanp4 and nanp7 but test Ab with nanp2,3 and 15?

RESPONSE: We have responded to this comment above. Briefly, we sought to identify the shortest number of repeats that could be recognised by antibodies and whether antibody specificity was associated with protection in RTS,S vaccinated children. For initial studies, we leveraged samples from a previous mouse vaccine study (vaccinated with four or nine NANP repeats) to evaluate antibody fine specificity and whether repeat length influenced antibody specificity or cross-reactivity. Please refer to the earlier response for a more detailed explanation.

Line 214 says nanp9 whereas line 213 says 7. What is "significant" igg?

RESPONSE: The text has been revised accordingly in page 11, line 263.

Line 227 "at higher concentrations...." - do they actually have concentrations? Than what?

RESPONSE: Yes, we tested the monoclonal antibodies at a two-fold dilution series between 0.001 and 2μg/ml as specified in the Methods (page 7, lines 156-157). We have revised the text in results section to clarify that mAb 317 demonstrated reactivity to $NANP_2$ when tested at a concentration of >0.25μg/mL on page 12 (line 278).

Line 231 " all, including... except" reword

RESPONSE: We have revised the text accordingly (page 12, line 279-280).

Line 234/5 "validated" suggest reword based on above comment.

RESPONSE: We have revised the text accordingly (page 12, line 285).

line 243 samples chosen for pool n=50 - what timepoints? How many malaria episodes (in what time period) did the vaccinated people have? How many malaria episodes (in what time period) did the control vaccinated people have?

RESPONSE: We evaluated the time to first event until 1.5 years post-vaccination as described in the Methods (page 8, lines 186-187 and page 10, lines 219-221), whereby 29% of children had at least one episode of malaria during this time. We randomly selected children who had a malaria episode (n=50) or remained malaria free (n=50) during this period of follow-up. From these we prepared a pool to evaluate antibody fine specificity before proceeding to test individual samples from the whole cohort. We have now included Supplementary Figure S1A which gives an overview of the study and the data in each figure to make this clearer.

line 245 what does "detectable" mean?

RESPONSE: We have revised the text accordingly to clarify that IgG specificity and cross-reactivity was observed to all peptides in the RTS,S pools compared to the control non-malaria vaccine group (defined by OD>0.2 at a 1/250 serum dilution factor) (page 13, lines 295-296).

line 248 - include p values

RESPONSE: It is not ideal to generate p-values when comparing two pooled samples. We have used the area under the curve to describe differences between the pools prior to testing individual samples. The studies with pools were not designed for formal statistical analysis. We subsequently tested samples from the whole cohort for antibodies and undertook formal statistical analysis of associations with protection which is much superior to an analysis of the pools.

line 250 - was avidity also investigated for NANP2, J1 as well as NANP15? If not, why not?

RESPONSE: The purpose of these assays was to determine whether children with higher avidity to the NANP-repeat region of CSP may have greater reactivity to $NANP_2$ and J1 peptides. This is because higher IgG affinity/avidity has been associated with antibody cross-reactivity in other systems. We have revised the wording to make this clear. Unfortunately, due to limited sample availability, we are unable to test antibody avidity to $NANP_2$ and J1 peptides. However, we do not expect this data would help interpret our findings.

Line 256 (and Fig 3 B) - please include mean/range of ages within age groups 12-24 and 24<60 months

RESPONSE: This information has been added to page 13, lines 315-316 and to figure legend of Fig3B.

line 257 "both tests" should this be "both antigens"?

RESPONSE: We have revised the text accordingly (page 13, line 317).

line 261 - correlation analysis - why was avidity only investigated for NANP15 and were these adjusted for multiple comparisons? Authors use 'avidity' and 'affinity' to mean the same thing here. I think it should be avidity both times.

RESPONSE: We have explained the reason for testing avidity to $NANP_{15}$ in the response above. We have revised the text to “avidity” (page 13, line 321). We have not adjusted p values for multiple comparisons since only a few comparisons were made and this is an exploratory analysis. We have reported rho values and p values to enable readers to interpret the strength and statistical significance of correlations.

line 269 - categorisation of "high" and "low" - based on what? At which timepoint?

RESPONSE: In our study, we tested samples collected 30 days after vaccine (month 3 timepoint in the study), which has now been specified in the Methods on page 8, lines 182-183 and figure legends. We defined children as high and low responders based on the median IgG response as specified on page 14, lines 329-330.

line 277 - please include the time to first episode for each group

RESPONSE: In Figure 3, we present Kaplan-Meier survival curves showing the time to first event in children given RTS,S (defined as a high or low antibody responder) and in children given the control vaccine. Further details on event rate can be found in the clinical trial (Alonso et al., Lancet 2004), which is referenced in the manuscript.

line 280 - what was the control vaccine?

RESPONSE: Children in the control vaccine group were given the pneumococcal conjugate, Haemophilus influenzae type b or paediatric hepatitis B vaccine depending on their age group. We have now specified these details in Methods on page 8, lines 178-182.

line 318 - suggest move to discussion

RESPONSE: We believe this text flows nicely in its current position.

line 327 - add ages of "young" children

RESPONSE: We have revised the text accordingly (page 17, line 389).

line 332 - suggest to amend "effectively" to "is able to differentiate" since this has not been used in a validation cohort

RESPONSE: We have revised the text accordingly (page 17, lines 393-394).

line 337 - given this paper relies upon the difference between length of sequence, please include some comment on the sequence used for functional assays and comparability to previous work showing complement and FcyR3 binding.

RESPONSE: We have revised the text accordingly to ensure the wording is clear and included further comments and future directions in the Discussion section (page 19, lines 456-460).

Line 338: The peptide sequence used to analyse functional antibody responses has not been used before. The references cited to support these functional activities as protective responses all use different antigens in their assays:

- Ref 26 (same as ref 43): Kurtovic et al 2024, use (NANP)15 and some of the C-term (separate assays)

- Ref 44: Behet et al 2018, use whole sporozoite assay—here they don't actually assess FcyR binding, they look at complement deposition and hepatocyte invasion.
- Ref 45: Behet et al 2014, also a whole sporozoite assay. Here they don't measure complement deposition or FcR binding, just the activity of IgG elicited by whole sporozoite immunization to inhibit hepatocyte traversal in vitro.

RESPONSE: We have revised the text accordingly to clarify what antigens have been used in past studies and suggested that future studies assess functional antibodies to additional epitopes (page 19, lines 456-460). The References to Behet et al., 2014 and 2018 have been removed from the manuscript specifically on page 15 (line 359) and page 17 (line 400).

line 361 - amend "was" to "has not been evaluated"

RESPONSE: We have revised the text accordingly on page 18, line 422.

Line 367: It would be interesting to use these outcomes in a predictive model to see if this statements 'identify children with no protective immunity' is reproducible.

RESPONSE: We agree this would be interesting and have specified in the manuscript that our assays need to be evaluated in other cohorts to further build an evidence base (page 21, line 501-506). A larger dataset from multiple trials may allow us to define threshold measures for protection or susceptibility.

line 395 - please comment on the fact that the most discriminitory group (high NANP2 and J1 responders) did not come out as a separate grouping. "longitudinal" - is this a longitudinal analysis? Do you mean longitudinal serological analysis or length of follow up for malaria episodes. This is confusing.

RESPONSE: We do not believe it is possible for the children to have low antibodies to $NANP_{15}$ but high antibodies to $NANP_2$ and J1. This is supported by our correlation data presented in Figure 5. As children were vaccinated with RTS,S containing multiple NANP repeats, we would not expect to find children with low/no antibodies to $NANP_{15}$ but high antibodies to NANP2 and J1. We refer to the study as longitudinal as the children were monitored for malaria for 1.5 years post-vaccination. We use the term longitudinal analysis

because we are including data from longitudinal follow-up over time. We believe our terminology is consistent with usage in the field.

line 400-402 - ages groups are NOT the same between RTSS vaccinated children in this study and R21 - please highlight this.

RESPONSE: We have revised the text accordingly (page 20, line 470-471).

line 408-409 - which markers of malaria exposure were used in ref 58?

RESPONSE: These were pre-vaccination IgG magnitudes to PfEXP1 and PfMSP2 antigens. We have revised the text accordingly (page 20, line 477-478).

Figures/Tables

Fig1 - please include the sequence use din functional assays

RESPONSE: We have revised the figure accordingly (page 30, line 765).

Fig 1 legend line 680 - "RTSS-like construct" would be good to reference work to evidence this

RESPONSE: We have specified the reference for this construct in methods page 7, lines 143-146.

Fig 2 - please add p values in each plot and specify timepoints samples were

RESPONSE: As explained above, it was not ideal to generate p-values when comparing two pooled samples in this analysis. We have used the area under the curve to describe differences between the pools prior to testing individual samples. Subsequently we tested all samples for reactivity to specific antigens and conducted a formal analysis of protective associations (Figure 4). In our study, we only evaluated samples collected 30 days after vaccination, as specified in the text and in all figure legends

Fig 3 B - age groups not comparable to R21 - cross ref line 400-402 in discussion

RESPONSE: We have revised the text accordingly (page 20, line 470-471).

Fig 3 B - please include mean/range of ages within age groups 12-24 and 24<60 months

RESPONSE: We have revised the figure legend text accordingly (page 33, lines 802-804).

Fig 4 - why are there only n=84 "controls"? How were they chosen? Are they representative of all controls? Line 251 says there were n=99. Again, how were the n=79 chosen for "high" and "low"? Line 276 says n=256 for "high" and n=220 for "low"

RESPONSE: Only children with complete follow-up data until 1.5 years post-vaccination were included in the protection analysis shown in Figure 4. We had included n=99 controls in our immunogenicity studies; only 84 had complete follow-up and could be included in the survival analysis. We have edited the figure legends to make this clear. We have further clarified the sample sizes in the results section to outline that out of 646 children with clinical data available, n=254 demonstrated high IgG to both $NANP_2$ and J1 and n=220 demonstrated low IgG to $NANP_2$ and J1 (page 14, lines 335-338). We measured functional responses to the Junction repeat peptide in a subset of RTS,S-vaccinated children (n=461) and of these, n=82 had high IgG to both $NANP_2$ and J1 and n=82 had low IgG to both peptides (page 15, lines 360-361) We note that the values of “n=256” and “n=79” were miswritten (should be “n=254” and “n=82”) and have updated this across the manuscript accordingly.

Fig 5B - please include the description for the clusters in the figure "High IgG to all 3", "high IgG to NANP15 and J1" etc.

RESPONSE: We have revised the figure accordingly (page 36, line 823).

Fig 5D - please include n's for this.

RESPONSE: We have revised the figure legend text accordingly (page 37, line 836).

supplementary methods

Why were (and what could be the consequences for this work) of using 3 different strains of mice?

RESPONSE: As previously mentioned, we leveraged samples from previous and independently conducted mouse vaccine studies. This is why there were some differences

in the way the studies were designed, such as the mouse breeds used. An advantage of using different vaccine types and different mice is that we can show some generalisability – we see variation in the specificity of antibodies and cross-reactivity of antibodies across the different mouse studies. We believe the main differences in antibody specificity arises from different construct designs.

Please include brief details of antigen manufacture

RESPONSE: Antigen were synthesised by Mimotopes (AU) or LifeTein (USA) and details are included in Methods (page 9, lines 199 and 206).

Please include brief details of merozoites

RESPONSE: We have provided details of merozoite culture and isolation (page 2, lines 52-57).

Please include catalogue details of reagents used in all assays, including functionality.

RESPONSE: We have added the catalogue numbers missing from the functional immunoassays section to page 3 (lines 62-65).

supplementary figures

Figure S1 - please include details of merozoite ODs (mean/SD/range) by exposure group

RESPONSE: This is now Figure S3. High and low exposure groups were defined by the median IgG response to merozoites (OD=0.220); the median values per exposure group have now been specified in the figure legend (page 9, lines 106-108).

Figure S2 - Why are authors only showing correlations between NANP15 avidity and NANP2 and J1 IgG? Where is NANP15 IGG and NANP15 avidity? Why are data on avidity to NANP2 and J1 not shown? Suggest to have a heatmap of all correlations and then adjust for multiple comparisons.

RESPONSE: This is now Figure S4. This point has been addressed in earlier questions from the reviewer, above. The purpose of these assays was to determine whether children with

higher avidity to the NANP-repeat region of CSP may have greater reactivity to $NANP_2$ and J1 peptides. This is because higher IgG affinity/avidity has been associated with antibody cross-reactivity in other systems. We have revised the wording to make this clear. Unfortunately, due to limited sample availability, we are unable to test antibody avidity to $NANP_2$ and J1 peptides. In any case, we do need believe that quantifying avidity to NANP2 and J1 peptides would address our aims. In response to the reviewer's comment, we have now included a correlation of IgG and avidity to $NANP_{15}$ in Supplementary Figure S4 and revised the text accordingly on page 10 (lines 111-115).

Figure S3 - it is not clear from the legend if this is the same study or not. The samples numbers are very different (n=461 and n=23 - how were these chosen if this is the same study?) What is the "non-malaria comparator vaccine" used here? Is this showing IgG responses to the "junction repeat" sequence that is used in the functional assays? If so, why is this not shown in a main figure? And why was it not done on all samples?

RESPONSE: This is now Figure S5. Yes, this data is from the same phase 2b clinical trial as the rest of the paper. We evaluated samples from one clinical trial, as specified in the Methods (page 8, lines 176-178). We tested all available samples from children in the RTS,S vaccine group (735 of 803; 92%) and a random selection of children in the control vaccine group (n=99) for IgG to $NANP_2$, $NANP_3$ and $NANP_{15}$ peptides. Details of the non-malaria control vaccine can be found in page 8 (lines 178-182). Unfortunately, due to limited sample volume, we were only able to test 461 and 23 samples from the RTS,S and control vaccine groups (respectively) for functional activity against the Junction-repeat peptide, as shown in Figure S5. We have provided the data here for further descriptive purposes and transparency, this figure is not a major point in the paper. We have revised the text to explain our sample size selection in the Methods section (page 9, lines 191-193) and provided Supplementary Figure S1A for further clarification.

Reviewer #3

This is a mixed method study that used both animal models and clinical participants from a randomised controlled trial study. I am not a animal model expert and only reviewed the statistical method for that part. The study design from the RCT appears appropriate. I have several questions about the statistical methods:

1. Please specify the rationale for using two-sample t-test in the animal model but the non-parametric Mann-Whitney U test for human participants.

RESPONSE: We performed parametric statistical testing for the preclinical studies as the mice were genetically identical and raised in the same pathogen-free conditions and therefore assumed to have a normal data distribution. In contrast, we performed non-parametric statistical testing for the clinical study as vaccine responses in African children has been previously shown to be heterogeneous and not normally distributed (e.g., Kurtovic et al., Lancet Microbe 2024).

2. Correlation coefficient was used to examine the relationship between IgG specificity to different peptides - this assumes linear dependence. Please specify if this assumption was examined. The scatterplot shown in Fig 5 is so noisy that it isn't clear if linear assumptions were appropriate.

RESPONSE: For correlations, we used Spearman's rho which does not assume linearity of the correlations and is appropriate. However, we have created some confusion by including a linear line of best fit on the graphs, which may not be appropriate. In the revised manuscript which have removed the lines from Figure 5 and Supplementary Figure S4.

3. Line 175 - shouldn't it be log rank *test*.

RESPONSE: We have revised the text accordingly (page 10, line 222).

4. Only age was adjusted in the Cox model - was only age thought to be the only confounder / prognostic factor? Were there any clinical factors that could affect antibody response as well as malaria risk? Please also specify if proportional hazard assumption holds.

RESPONSE: We did adjust the analysis for age because age has been associated with vaccine efficacy and we found that age was associated with the vaccine response (see Figure 3). We considered sex, *a priori*, but we found no association with antibody responses (Figure 3). Of the clinical data available, only age was significant associated with antibody responses and malaria risk. Therefore, age was the only confounded adjusted for in our final model, consistent with analysis we have previously published on this cohort (Kurtovic et al., Lancet Microbe 2024).

Yes, we evaluated the proportional hazards assumption test (p>0.05) and can confirm the assumptions have been met. We have clarified this in the methods (page 10, lines 222-228).

5. Line 181 - 'cluster analysis' instead of 'clustering'?

RESPONSE: We have revised the text accordingly (page 10, line 230).

Reviewer #4

Hysa et al report on the important finding of antibody fine specificity to minimal epitopes as correlates for the RTS,S malaria vaccine in young children. The strengths of the manuscript include the important area of study, unique clinical cohort, and experimental approach to assess binding specificity. Overall this study reports novel information on antibody specificity and subgroup analyses to better understand antibody correlates in young children. However, the correlates analyses lacks a pre-specified analytical plan, the link between the preclinical and pooled data with he clinical trial is not clear; and the overall analysis lacked information on other immune features, demographics or other confounding variables.

RESPONSE: We have addressed the reviewer's comments with several revisions and additions. We have now included a study overview figure as Supplementary Figure S1, which summarises our study approach and the data shown in each figure. We believe this makes it clearer for the reader how the different experiments, analyses, and data types are linked. We have also made various edits and revisions throughout the text to make it clearer. We did have a pre-specified analysis plan and we have made revisions in the manuscript to make this clearer. In Supplementary Figure S1B, we have included the statistical analysis framework we used to help address this point. We evaluated the association between antibody responses and time to first event (clinical malaria), which is the analysis approach used in the original trial (Alonso et al 2004), and is a method that has been previously used to determine potential immunological correlates of protection in studies of natural and vaccine immunity (e.g., Reiling et al., Nat Commun 2019; Olotu et al., Lancet Infect Dis 2011).

We adjusted our analysis for age in our analysis, as this was the only parameter shown to be associated with antibody responses and malaria risk, we have now further explained this in our methods (page 10, lines 222-228).

Specific comments:

It is not clear how the inclusion of the preclinical model informs the evaluation of the presented evaluation of the human trial. It is noted that the mouse vaccination study 'validated antibody specificity and cross-reactivity'; yet it is not clear how this was done given the substantial differences between murine and human antibodies.

RESPONSE:

We included mouse vaccination studies to initially evaluate responses to our peptides and confirm our approach for quantifying epitope-specific responses since these are not well established in studies of malaria vaccine immunology. As mentioned in the manuscript, we used mouse vaccine studies to evaluate responses without the "potential confounding effect of prior exposure to malaria which is present in vaccinated children from endemic regions" (page 11, lines 245-247). We included mice vaccinated with full-length CSP, the RTS,S construct and just NANP-repeats which has not been trialled in humans before; using mouse studies enabled us to evaluate response to these different antigens and better understand what determines responses. RTS,S includes other CSP regions within it, and therefore, the inclusion of NANP-vaccinated mice provided a way to measure responses specifically induced by the central repeat region of CSP. We acknowledge that the mouse vaccination data is not the major focus of our paper, but we believe the data are helpful and valuable to include for the reasons outlined.

The text mentions validated assays were utilized, but information on the validation (repeatability, robustness, linearity etc) is not provided.

RESPONSE: For clarity, we have removed the term 'validated' because it is used in specific contexts on page 6 (line 131), page 11 (line 244), page 12 (line 285) and page 17 (line 404-405).

For the statistical and CoP analyses, is there a threshold of Ab specificities that are critical?

RESPONSE: In the field, there is currently no defined antibody threshold that correlates with protection. Therefore, we could not include such threshold in our analysis. In the future, generating similar data from other trials would help create a dataset that would better enable

the identification of potential thresholds or cut-offs for protection or defined levels of vaccine efficacy.
