# Supplementary material for: Antibody fine specificity correlates with protection from malaria for the RTS,S vaccine in young African children: A post hoc analysis of a phase IIb randomised controlled trial
Source: PLoS Med. 2026 May 14;23(5):e1004877. doi: 10.1371/journal.pmed.1004877 (PMC13186370; doi:10.1371/journal.pmed.1004877)
Supplement: Attachment — Submitted filename: Editor and reviewer responses_Hysa et al_April 2026.pdf [file pmed.1004877.s007.pdf]

Antibody fine specificity correlates with protection from malaria for the RTS,S vaccine in young African children: analysis of a phase IIb randomised control trial.

Hysa, Opi, Waterhouse, Chishimba, Horton, Kingston, Netter, Wetzel, Piontek, Feng, Sacarlal, Dobaño, Kurtovic and Beeson.

RESPONSE TO EDITOR AND REVIEWER COMMENTS

Please note that all page and line numbers refer to the revised <u>clean</u> manuscript file and supplementary file which are now two separate documents.

<u>Requests from Editors: Please provide a rebuttal clarifying how you have addressed these points.</u>

Please review your text for claims of novelty or primacy (e.g. 'for the first time') and remove this language. In addition, please check that any use of statistical terms (such as trend or significant) are supported by the data, and if not please remove them.
Text revised accordingly: page 1 (line 1), page 19 (line 432), page 21 (line 472).

Please provide URLs for all funders and provide grant numbers for all relevant instances.
Provided all where available and relevant.

Please revise the Data Availability Statement to remove the 'reasonable request' phrasing and specify all requirements or restrictions to data access.
Updated, pages 41-42 (lines 919-925).

Please use RTS,S/AS01 at first mention of the vaccine in the Abstract and follow it with (RTS,S).
Updated, page 2 (line 28).

Similarly, please use the full name of R21 at first mention in the Introduction.
Updated, page 6 (line 91).

Line 30, revise "Vaccine-induced CSP mechanisms of immunity and correlates of protection are not well defined..." to "Mechanisms of immunity and correlates of protection for RTS, S are not well defined..."
Updated, page 2, (lines 31-33).

Line 36: "We evaluated responses" should be "We evaluated antibody responses".
Please also move ' monoclonal antibodies ' to the end of the sentence and add their specificity. E.g. "(n=735), as well as CSP-specific monoclonal antibodies."
Updated, page 2 (line 36-37).

If the clinical trial is named, please add the name. Named “RTS,S phase 2 clinical trial” throughout manuscript.
Text revised accordingly: page 1 (line 2), page 2 (line 38, line 45), page 20 (line 429).

Please start the paragraph on line 38 with a one sentence discussion of the results from the mouse studies and analyses of mAbs. As written, the Abstract does not mention the findings in the preclinical studies or rationale for their inclusion.
Updated, page 2 (lines 40-44).

Line 38: Please refer to the name of the clinical trial here or refer to it as the phase 2 trial and revise as "In analyzing antibody responses in the phase 2 trial, we found..."
Updated, page 2 (line 44-45).

Please clarify in the Abstract that you performed a post hoc analysis of clinical trial samples.
Updated, page 2 (line 44).

* In the last sentence of the Abstract Methods and Findings section, please describe the main limitation(s) of the study's methodology.
Updated, page 3 (lines 52-54).

* Please include statistics in the Abstract and more specifics about the key findings.
Updated, page 2 (lines 46-51).

* Please use the active voice throughout.
Text revised accordingly: page 14 (line 328).

* The Author Summary also makes no mention of the mouse studies. Please add a bullet point pertaining to them, explaining why they are included in this study.
Updated, page 4 (lines 72-73).

* Please temper the conclusions in the Author Summary, final bullet point. The study identifies candidate correlates of protection. Please reframe accordingly. Please explain that more studies are needed to independently validate and confirm the association and to test whether eliciting these antibodies would confer protection against malaria.
Updated, page 5 (lines 81-86).

* In the author summary, in the final bullet point of 'What Do These Findings Mean?', please include the main limitations of the study in non-technical language.
Updated, page 5 (lines 85-86).

The statements regarding the clinical trial in the Acknowledgments should be moved to a separate Disclaimer subheading (lines 864-867).
Updated, page 41 (lines 905-910).

In Fig 5c, please confirm that the P values comparing NANP15 and J1 are correct in all 4 clusters.
Updated, page 39 (line 880), mistakenly swapped around the p-values.

Please add a statement to the Methods that you have not corrected for multiple testing.
Updated, page 13 (lines 276-277).

* Authors DW and MP are employees of ARTES Biotechnology Gmbh. Please therefore revise the competing interests statement.
Updated, page 41 (lines 908-910).

* Please also add this statement to the manuscript's Competing Interests: "[Initials] is an Academic Editor on PLOS Medicine's editorial board."
Updated, page 41 (lines 908-910).

* Please revise your title to comply with PLOS Medicine's style. Your title must be nondeclarative and not a question. It should begin with main concept if possible. "Effect of" should be used only if causality can be inferred, i.e., for an RCT. Please place the study design ("A randomized controlled trial," "A retrospective study," "A modelling study," etc.) in the subtitle (ie, after a colon).
Updated, page 1 (lines 1-3).

* Please ensure that the Introduction ends with a clear description of the study question or hypothesis.
Included, page 8 (lines 149-151).

* Please ensure that all abbreviations are defined at first use throughout the text. Text revised accordingly: page 2 (line 40), page 7 (line 115).

* Please confirm that all numbers presented in the abstract are present and identical to numbers presented in the main manuscript text.
Confirmed.

Line 266: please suggest a possible reason.
Included in the Discussion section, page 21 (lines 454-457): "The observed cross-reactivity may be due to shared amino acids between NANP, NVDP and NPDP motifs.".

Lines 297, 300: Are the children in this trial from a single country? If so, please identify it.
Updated, page 15 (line 329).

Line 306, please correct grammar.
Updated, page 16 (lines 339-340).

Line 310: Please qualify explore (i.e. explore what?)
Updated, page 16 (line 342).

Line 310: Please refer to testing samples from children, not to testing children, as written.
Updated, page 16 (line 343).

Line 428: Please revise 'which were' to 'which was'.
Updated, page 21 (line 460).

Line 498-500: sentence uses valuable twice. Please revise.
Updated, page 23 (line 528).

"* Statistical reporting: Please revise throughout the manuscript, including tables and figures.
- Please report statistical information as follows to improve clarity for the reader ""22% (95% CI [13,28]; p</=)"".

- Please separate upper and lower bounds with commas instead of hyphens as the latter can be confused with reporting of negative values.
- Please repeat statistical definitions (HR, CI etc.) for each set of parentheses."

Updated on page 18 (lines 406-407); in Figure 4B, page 37 (line 859) and in Supplementary Table S2, page 5 (line 81).

"* PLOS defines the "minimal data set" to consist of the data set used to reach the conclusions drawn in the manuscript with related metadata and methods, and any additional data required to replicate the reported study findings in their entirety. Authors do not need to submit their entire data set, or the raw data collected during an investigation.

Please submit the following data:
The values behind the means, standard deviations and other measures reported;
The values used to build graphs;
The points extracted from images for analysis."

Minimal data set supplied.

* Please define all elements of box plots in the figure caption - center line, box limits and whiskers.

Updated, Figure 1 legend, page 34 (lines 828-829, 833-834); Figure 3 legend, page 36 (lines 857-858); Figure 4 legend, page 38 (lines 878-879); Figure 5 legend, page 40 (lines 894-895); Supplementary Figure 2 legend, page 8 (line 101); Supplementary Figure 3 legend, page 9 (lines 111-112); Supplementary Figure 5 legend, page 11 (lines 126-127).

* Please ensure that where relevant figures include 95% CIs.

Updated in Figure 4B, page 37 (line 859).

* Please show graph axes beginning at zero. If this is not possible, please show a break in the axis.

Updated in Figure 4A, page 37 (line 859).

* When a p value is given, please specify the statistical test used to determine it in the legend.

Updated, page 37 (line 867), page 40 (line 884).

* Where data points are discrete, please ensure that they are depicted in the figures as discrete data and not as a continuous line.

Confirmed.

* In the Kaplan-Meier curve(s) please provide the number at risk for each time interval. Information on this has been provided in the Figure or Figure legend where relevant, page 37 (lines 864-866 and lines 870-871).

*Please clearly state in the Methods why the peptides used in the mouse studies differ from those in the analysis of human samples. While you have responded to the referees about this issue, it is not sufficiently explained in the manuscript.
Updated, page 9 (lines 164-166 and line 171-172).

* Please report your study according to the ARRIVE guidelines and checklist https://urldefense.com/v3/__http://www.nc3rs.org.uk/arrive-guidelines__;!!G5ONXFxL7-lpCg!aRHzkToJaQ0rlXdyRzzmqV0vALNagbXsrdVsKXqsjwCsHgFR1puT4xxKJNF4Tq0SfYfqeizxeBFSQBUfOJw$ In the checklist please include sufficient text excerpted from the manuscript to explain how you accomplished all applicable items.
Completed checklist provided as supplementary file.

* Please also include a completed STROBE checklist. Please add the following statement, or similar, to the Methods: ""This study is reported as per the Strengthening the Reporting of Observational Studies in Epidemiology (STROBE) guideline (S1 Checklist)."" The STROBE guideline can be found here: https://urldefense.com/v3/__http://www.equator-network.org/reporting-guidelines/strobe/__;!!G5ONXFxL7-lpCg!aRHzkToJaQ0rlXdyRzzmqV0vALNagbXsrdVsKXqsjwCsHgFR1puT4xxKJNF4Tq0SfYfqeizxeBFSqHlc_kc$ When completing the checklist, please use section and paragraph numbers, rather than page numbers."
Completed checklist provided as supplementary file and mentioned on page 11 (lines 217-219).

"* Did your study have a prospective protocol or analysis plan? Please state this (either way) early in the Methods section.

a) If a prospective analysis plan (from your funding proposal, IRB or other ethics committee submission, study protocol, or other planning document written before analyzing the data) was used in designing the study, please include the relevant prospectively written document with your revised manuscript as a Supporting Information file to be published alongside your study, and cite it in the Methods section. A legend for this file should be included at the end of your manuscript.

b) If no such document exists, please make sure that the Methods section transparently describes when analyses were planned, and when/why any data-driven changes to analyses took place.

c) In either case, changes in the analysis-- including those made in response to peer review comments-- should be identified as such in the Methods section of the paper, with rationale."
We have further expanded our description of the statistical analysis plan and approach in the Methods section to address these points on page 12-13 (lines 258-266).

* Your study is observational and therefore causality cannot be inferred. Please remove language that implies causality and refer to associations instead.
Confirmed.

* For all observational studies, in the manuscript text, please indicate: (1) the specific hypotheses you intended to test, (2) the analytical methods by which you planned to test them, (3) the analyses you actually performed, and (4) when reported analyses differ from those that were planned, transparent explanations for differences that affect the reliability of the study's results. If a reported analysis was performed based on an interesting but unanticipated pattern in the data, please be clear that the analysis was data-driven.
Text has been added to the methods to specifically state the hypothesis being tested and ensure that the statistical analysis approach was clearly explained on page2 12-13 (lines 258-266).

PLOS has an 'Inclusivity in Global Research' policy which aims to promote collaboration and inclusivity in global health research. You are required to complete PLOS' questionnaire on inclusivity in global research and submit it with your revised paper. The policy and questionnaire can be found at https://urldefense.com/v3/__https://journals.plos.org/plosone/s/best-practices-in-research-reporting__;!!G5ONXFxL7-lpCg!aRHzkToJaQ0rlXdyRzzmqV0vALNagbXsrdVsKXqsjwCsHgFR1puT4xxKJNF4Tq0SfYfqeizxeBFSy7l7RWs$.
Completed document provided as supplementary file and mentioned on page 11 (lines 159-160).

Comments from Reviewers

Reviewer #1: I thank the authors for their very detailed and helpful responses to my comments and to those of the other reviewers on this important paper.

Reviewer #2: All edits and suggestions addressed.

Reviewer #3: Thank you for addressing my comments but the responses raise a couple more questions:

1. Please ensure the rationale for selecting tests (e.g. parametric vs. non-parametric tests) are communicated through the text.

Updated, page 12 (lines 240-246).

2. Thank you for clarifying the use of Spearman's correlation. It does assumes / examine only monotonic relationship. What i was wondering was if the relationship is more complicated than that. E.g. in Figure 5A, left panel, at a-axis around 2, the cluster of data points around y=0.5. I don't think there'd be any statistical methods that could test this because of the sample size is so small at that region - similarly these may as well be just random noise. However, I do think it is important to make sure the assumptions (i.e. monotonic relationship) is reported.
In response to these comments, we have updated the methods to state that we assessed monotonic relationships, page 12 (line 248).

3. From the response to my question 4 in the first review, the authors implied that the selection of covariates are based on statistical significance which is not what is being recommended currently. Statistical inference for aetiological study should adjust based on a priori knowledge (common causes of exposure and outcome) rather than based on empirical p-values from univariate analysis. If the authors do not wish to reanalyse, please do acknowledge / discuss this.
There may have been some misunderstanding from the reviewer here, or we may not have communicated our approach clearly in our previous response. In the manuscript we state that age and sex were included as potential confounders 'a priori' page 12-13 (lines 258-266) which is aligned with the reviewer's preference. Previous analyses of this clinical trial had not identified other confounders, nor did we identify other confounders. We have made some minor revisions and expanded the explanation in the methods section to ensure our approach is clear on page 12-13 (lines 258-266).
